# Adult growth hormone deficiency treatment with a combination of growth hormone and insulin-like growth factor-1 resulting in elevated sustainable insulin-like growth factor-1 and insulin-like growth factor binding protein 3 plasma levels: a case report

**DOI:** 10.1186/1752-1947-4-305

**Published:** 2010-09-15

**Authors:** Eric R Braverman, Abdalla Bowirrat, Uma J Damle, Swetha Yeldandi, Thomas JH Chen, Margaret Madigan, Mallory Kerner, Stanley X Huang, Stella Savarimuthu, Kenneth Blum

**Affiliations:** 1 Department of Neurological Surgery, Weill Cornell College of Medicine, New York, NY, USA; 2 Clinical Neuroscience and Population Genetics, Ziv Government Medical Center, Safed, Israel; 3 Department of Clinical Neurology, PATH Foundation NY, NewYork, NY, USA; 4 Department of Occupational Safety and Health, Chang Jung Christian University, Taiwan, R. O. C; 5 Department of Nutrigenomics Reward Deficiency Solutions, LLC, San Diego, CA, USA; 6 Department of Psychiatry, University of Florida College of Medicine, Gainesville, FL, USA

## Abstract

**Introduction:**

Adult Growth hormone Deficiency is a well known phenomenon effecting both males and females. Adult Growth Hormone Deficiency is marked by a number of neuropsychiatric, cognitive performance, cardiac, metabolic, muscular, and bone symptoms and clinical features. There is no known standardized acceptable therapeutic modality to treat this condition. A recent meta-analysis found that after 16 years of Growth Hormone replacement therapy a large proportion of the patients still had Growth Hormone associated symptoms especially related to executive functioning. A major goal is to increase plasma levels of both insulin-like growth factor (insulin-like growth factor-1) and insulin-like growth factor binding protein 3.

**Case Presentation:**

We report a case of a 45-year-old caucasian woman with early ovarian failure for 2 years and amenorrhea since the age of 43, who presented with Adult Growth Hormone Deficiency and an IGF-1 of 126 ng/mL. Since her insulin-like growth factor-1 was lowest at 81 ng/mL, she was started on insulin-like growth factor-1 Increlex at 0.2 mg at bedtime, which immediately raised her insulin-like growth factor-1 levels to 130 ng/mL within 1 month, and 193 ng/mL, 249 ng/mL, and 357 ng/mL, after 3, 4, and 5 months, respectively, thereafter. Her insulin-like growth factor binding protein 3 continued to decrease. It was at this point when we added back the Growth Hormone and increased her Increlex dosage to 1.3 - 1.5 mg that her insulin-like growth factor binding protein 3 began to increase.

**Conclusion:**

It appears that in some patients with Adult Growth Hormone Deficiency, insulin-like growth factor-1 elevation is resistant to direct Growth Hormone treatment. Furthermore, the binding protein may not rise with insulin-like growth factor-1. However, a combination of Growth Hormone and insulin-like growth factor-1 treatment may be a solution.

## Introduction

Adult Growth Hormone Deficiency (AGHD) is a well known phenomenon effecting both males and females. AGHD is marked by a number of neuropsychiatric, cognitive performance, cardiac, metabolic, muscular, and bone symptoms and clinical features [1]. In one study it was found that after one year of growth hormone (GH) replacement therapy in ovarian cancer patients among others, the mean value of all tumor markers remained within the normal range and there was no significant increase within the normal range either. This finding was accompanied by an increase of insulin-like growth factor (IGF-1) plasma levels [2].

However, the use of GH replacement therapy has not always resulted in consistent increases of IGF-1 in AGHD patients. The mechanism of this inconsistency has been studied by De Gennaro *et al. *[3]. In a series of experiments they investigated the effect of thyroid hormone deficiency and GH treatment on hypothalamic GH-releasing hormone (GHRH)/somatostatin (SS) concentrations, GHRH/SS mRNA levels, and plasma GH and somatomedin-C (IGF-1) concentrations in rats made deficient by exposing dams with propylthiouracil in the drinking water since the day of parturition. Treatment of hypothyroid rats with GH for 14 days completely restored hypothalamic GHRH content and reversed the increase in GHRH mRNA, but did not alter plasma IGF-1 concentrations. These data indicate that, in hypothyroid rats, the changes in hypothalamic GHRH content and gene expression are due to the GH deficiency ensuing from the hypothyroid state. Failure of the GH treatment to increase plasma IGF-1 indicates that the feedback regulation on GHRH neurons is operated by circulating GH and/or perhaps tissue but not plasma IGF-1 concentrations. Presence of low plasma IGF-1 concentrations would be directly related to thyroid hormone deficiency in this case but consistent methods for augmenting its levels in AGHD are unfortunately lacking. There is no known standardized acceptable therapeutic modality to treat this condition.

While there is a plethora of literature from meta-analysis showing the medical benefits of GH replacement therapy in cardiovascular disease, cancer risk, HIV, bone mineral density, fibromyalgia, head trauma, brain processing speed among others [3-10], there remains controversy as to what actually constitutes AGHD.

Previously, a number of hormonal test levels had to be lowered or raised as new research supported specific level changes (that is, PTH, TSH). In adults, a similar problem has arisen for an appropriate reference standard for IGF-1 and AGHD. Falletti *et al. *[3] in their meta-analysis suggested that the notion of IGF-1 plasma levels to be an independent diagnostic marker in AGHD is controversial. Many endocrinology studies link low IGF-1 plasma levels with low levels of other anterior pituitary hormones, that is, LH, FSH, and TSH [11]. It is noteworthy that low IGF-1 between 84 and 100 u/l with other anterior pituitary deficiencies has been considered sufficient evidence for GH therapy. However, we recently reported that IGF-1 deficiencies occur independently of comorbid deficiencies of LH, FSH, and TSH [12].

We report on a case where a woman with early ovarian failure, accompanied by seemingly low IGF-1 plasma levels, had severe comorbid symptoms which worsened as her IGF-1 levels continued to fall independent of anterior pituitary hormone deficiency assessment. We show that a combination of GH and IGF-1 replacement therapy to increase both IGF-1 and IGF-BP3 plasma levels may indeed be an important novel therapeutic modality to treat AGHD independent of targeting anterior pituitary hormone deficiencies.

## Case presentation

A 45-year-old Caucasian woman with early ovarian failure of two years duration came to our clinic. She was diagnosed with AGHD with a IGF-1 plasma level of 126 ng/mL. At entrance to our program she was on Human Growth Hormone at a dosage of 0.2 mg. Follow-up blood analysis revealed that instead of rising, her IGF-1 level continued to fall and she felt her symptoms worsening. These symptoms included abdominal distention and bloating, sleeping problems, depression, nervousness, loss of muscle mass, decreased bone density and weight change (increase). Interestingly, her IGF-BP3 level was also low.

**Figure 1:**
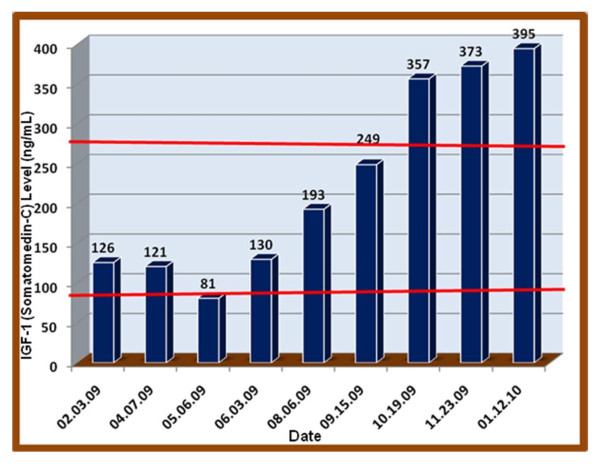
**IGF-1 (Somatomedin-C)**. Reference range: 94 to 267 ng/mL

During the course of treatment, following treatment with Norditropin^® ^(somatropin (rDNA origin)) by injection at 0.45 mg, her GH level continued to drop. The dosage level was increased to 0.6 to 0.7 mg and she experienced rapid weight gain. At that point the dosage level was decreased to 0.5 mg. Subsequent to this treatment we determined that while her IGF-1 level increased, her IGF-BP3 level decreased and her symptoms worsened, especially the rapid weight gain. The Norditropin^® ^regimen was terminated on 6 August 2009. On 6 May 2009, we found that her IGF-1 plasma level was at a low level of 81 ng/ml. (see Figure 1).

**Figure 2:**
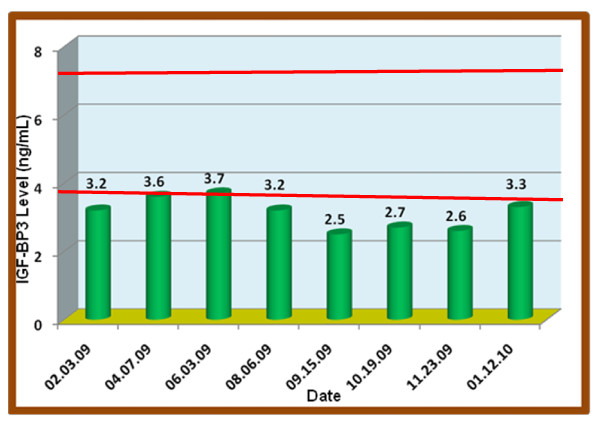
**IGF-BP3**. Reference range: 3.4 to 7.0 ng/mL

At this juncture one of us (ERB) considered the use of Increlex^® ^(a mecasermin (rDNA origin) injection). This agent is an aqueous solution for injection containing human insulin-like growth factor-1 (rhIGF-1) produced by recombinant DNA technology. IGF-1 consists of 70 amino acids in a single chain with three intramolecular disulfide bridges and a molecular weight of 7649 daltons. The amino acid sequence of the product is identical to that of endogenous human IGF-1. The rhIGF-1 protein is synthesized in bacteria (*Escherichia coli*) that have been modified by the addition of the gene for human IGF-1. This agent was started at 0.2 mg at bedtime which we found to raise her IGF-1 level to 130 ng/ml within one month and, as observed in Figure 1, increased by 6 August 2009 to 193 ng/ml, then to 249 ng/ml on 5 September 2009, and finally on 1 January 2010 it was at a high of 395 ng/ml. Surprisingly her IGF-BP3 plasma levels continued to decrease to 2.5 ng/ml. On 23 November 2009 we re-administered Norditropin^® ^in combination with an increased amount of Increlex^® ^between 1.3 and 1.5 mg. Subsequent to this treatment while her IGF-1 level was maintained at a high level (373 to 395 ng/ml) her IGF-BP3 began to increase to a peak concentration of 3.3 ng/ml as can be observed in Figure 2.

Following this novel combined treatment we observed an improvement in both physiological and neuropsychiatric symptoms.

## Discussion

Adult Growth Hormone Deficiency (AGHD) is marked by a number of neuropsychiatric, cardiac, metabolic, muscular, bone symptoms and clinical features. The most common of these are increased body fat (particularly abdominal fat), decreased lean body mass (including muscle) and functional strength, thin skin and cool extremities, decreased psychological well-being and energy, reduced bone density, an increase in c-reactive protein, low-density lipoprotein (LDL), fibrinogen, and plasminogen activator inhibitor-1 (PAI-1), a decrease in high-density lipoprotein (HDL), decreased insulin sensitivity, and decreased quality of life [13].

It appears that in some patients with AGHD, IGF-1 elevation is resistant to direct GH treatment. The interaction of IGF-1 and IGF-BP3 is quite complex but seems to involve the integrity of the GH receptor. Previous work from Wilson's laboratory has shown that the constant subcutaneous infusion of IGF-1 to monkeys with normal pituitary glands results in a sustained elevation in circulating concentrations of IGF-BP3, whereas the acute administration of IGF-1 to monkeys pretreated with a GH receptor antagonist produces a brief, but significant, elevation in serum IGF-BP3. Experiments from Wilson's group indicate that IGF-1 administration during GH receptor antagonism restores circulating levels of IGF-BP3. It remains to be determined whether IGF-1 directly affects hepatic synthesis and secretion of IGF-BP3 [14]. Therefore the use of IGF-1 alone may not be enough to raise IGF-BP3 levels but in combination with GH receptor agonistic activity it may induce the increases we observed herein.

Finally, we reported earlier from our laboratory [13] on the benefits of increasing low IGF-1 plasma levels to normal values. These benefits, which are consistent with literature findings [15], include an increase of IGF-1 levels to the high normal range; reverses of CMD and BMD from 138.1 to 279.4; fibromyalgia patients showed significant improvement with IGF-1 level of 98.6 to 173.3; brain processing speed and memory improved with IGF-1 levels of 150 to 250; 100-point increase of IGF-1 levels associated with seven-point increases in I.Q.; an IGF-1 elevation of 74.0 to 362.6 in head trauma patients; improved anxiety, depression, and short/long term memory; improvement in cognitive function from 135 to 213; reduced carotid intimal media thickness from 51.8 to 234.4; improvement in insulin sensitivity IGF-1 levels of 103.5 to 231.1; and reduction of abdominal fat accumulation occurring with the increase of IGF-1 from 146 to 267.

## Conclusion

The IGF-BP3 binding protein may not rise with IGF-1 treatment alone. The need for both IGF-1 and IGF-BP3 plasma levels to rise especially in cases with AGHD as seen in the case presented herein is adequately supported in the literature. Impairment of this complex system may be overcome by the combination of both GH (using Norditropin^®^) and IGF-1 (using Increlex^®^) and must await further confirmation in a large population.

## Competing interests

The authors declare that they have no competing interests.

## Consent

Written informed consent was obtained from the patient for publication of this case report and accompanying images. A copy of the written consent is available for review by the Editor-in-Chief of this journal.

## Authors' contributions

All authors actively participated by writing the article and developing graphics and literature citations. All authors read and approved the final manuscript.
